# Crystal structure of (4-chloro­phen­yl)[2-(10-hy­droxy­phenanthren-9-yl)phenanthro[9,10-*b*]furan-3-yl]methanone

**DOI:** 10.1107/S1600536814022338

**Published:** 2014-10-18

**Authors:** L. U. Sajitha, M. Sithambaresan, Jomon P. Jacob, M. R. Prathapachandra Kurup

**Affiliations:** a Department of Applied Chemistry, Cochin University of Science and Technology, Kochi 682 022, India; b Department of Chemistry, Faculty of Science, Eastern University, Chenkalady, Sri Lanka

**Keywords:** crystal structure, furan, phenanthrene, hydrogen bonding

## Abstract

In the title compound, C_37_H_21_ClO_3_, the dihedral angle between the two phenanthrene moieties is 57.79 (5)°. The furan and one of the phenanthrene groups are fused in an almost coplanar arrangement [dihedral angle = 5.14 (8)°] and the furan unit makes dihedral angles of 70.27 (11) and 57.58 (8)° with the planes of the phenyl and the second phenanthrene group, respectively. In the crystal, neighbouring mol­ecules are connected *via* two inter­molecular hydrogen-bonding inter­actions (O—H⋯O and C—H⋯O) towards the carbonyl O atom with donor–acceptor distances of 2.824 (2) and 3.277 (3) Å, creating an inversion dimer. A non-classical C—H⋯Cl inter­action [3.564 (2) Å] and three C—H⋯π inter­actions, with C⋯π distances of 3.709 (3), 3.745 (2) and 3.628 (3) Å, connect the mol­ecules, forming a three-dimensional supra­molecular architecture in the solid state.

## Abstract 2

Mol­ecules of the title compound are arranged in the solid state in a three-dimensional supra­molecular architecture *via* inter­molecular O—H⋯O and C—H⋯O hydrogen bonding and through C—H⋯π inter­actions.

## Chemical context   

Furan and its derivatives have in recent years again attracted the attention of researchers from various areas of chemistry (Uchuskin *et al.*, 2014 ▶; Liu *et al.*, 2013 ▶). The di­hydro­furan core framework was identified in many natural products and in drugs with remarkable biological activities (Michael, 2000 ▶; Lipshutz, 1986 ▶), inspiring the development of new synthetic methods for the construction of functionalized furans (Singh & Batra, 2008 ▶; Snider, 1996 ▶; Ranu *et al.*, 2008 ▶; Redon *et al.*, 2008 ▶; Adamo *et al.*, 2009 ▶). As for most organic syntheses, furans are often synthesized in stepwise sequences. However, it is much more efficient if one can form several bonds in one sequence without isolating the inter­mediates, changing the reaction conditions, or adding reagents (Tietze & Beifuss, 1993 ▶). This type of reaction, commonly termed a domino reaction (Muthusaravanan *et al.*, 2013 ▶; Kadzimirsz *et al.*, 2008 ▶; Criado *et al.*, 2013 ▶) would allow a substantial reduction of waste compared to stepwise reactions. The amount of solvents, reagents, adsorbents, and energy would also be dramatically decreased. 
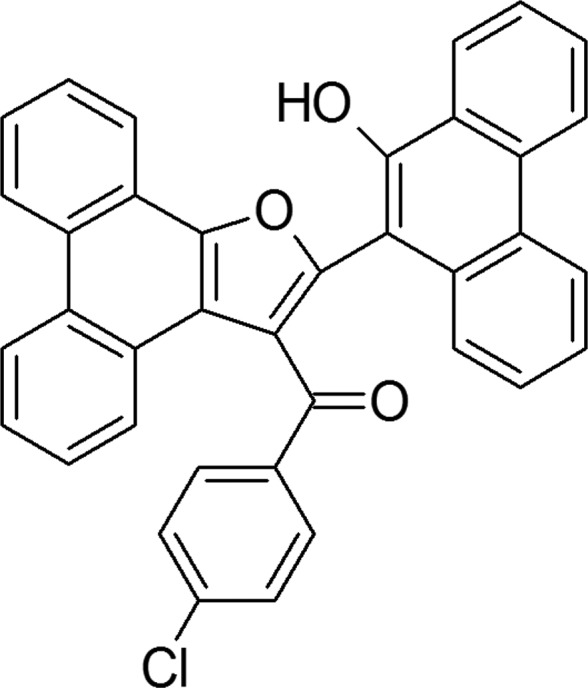


The title compound of this report has been obtained using such a domino reaction. Using a tandem Michael–aldol reaction of phenanthrene­quinone (1) with 4-chloro­aceto­phenone (2) we were able to obtain the highly substituted furan (3) and the 3(2*H*)-furan­one (4) (Jacob *et al.*, 2005 ▶) in one simple multicomponent reaction.

## Structural commentary   

**Figure 1:**
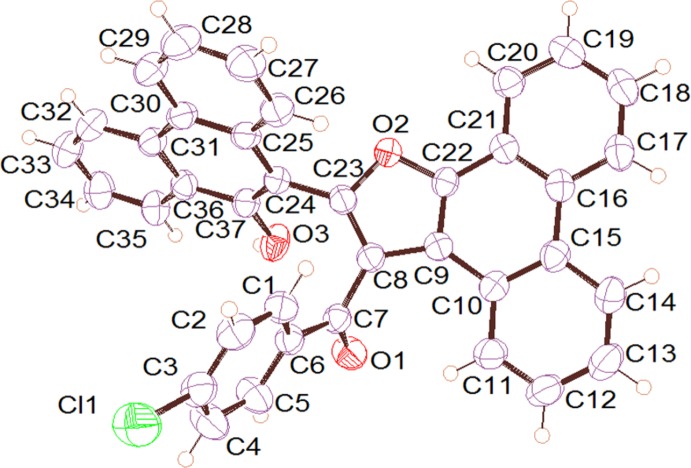
View of the title compound (3) with atom labelling. Displacement ellipsoids are drawn at the 50% probability level.

In the title compound, (3), the two phenanthrene moieties make a dihedral angle of 57.79 (5)°, while one of the phenanthrene moieties is fused together with the furan ring in an almost coplanar arrangement [5.14 (8)°] (Fig. 1 ▶). The central furan ring makes dihedral angles of 70.27 (11) and 57.58 (8)° with the phenyl ring and the other phenanthrene moieties, respectively. These two attached rings are twisted so that the C=O oxygen atom points away from the phenanthrene ring. This conformation is stabilized by intra­molecular hydrogen bonds between the H atoms attached to atoms C11 and C26 towards O1 and O2, respectively (see Table 1 ▶ for numerical values).

## Supra­molecular features   

**Figure 2:**
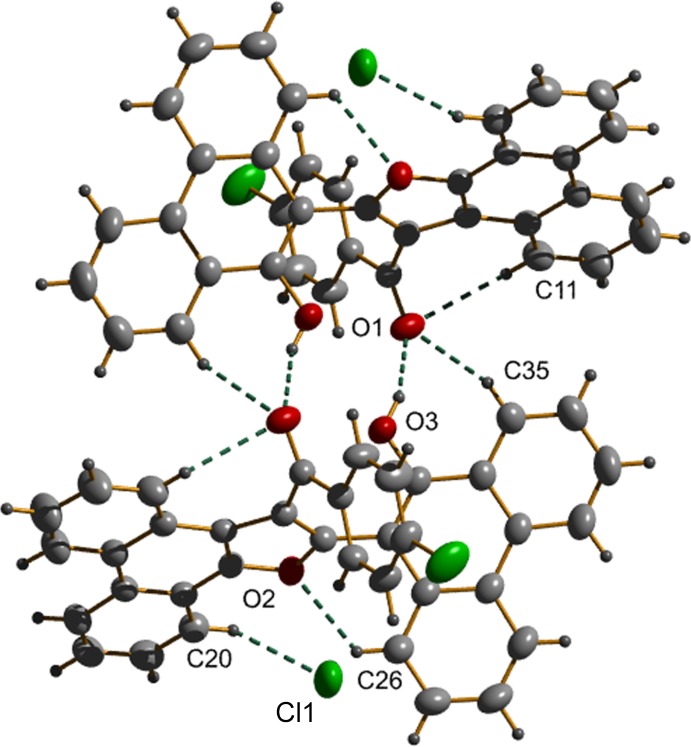
Hydrogen-bonding inter­actions found in the title compound (see Table 1 ▶ for details).

**Figure 3:**
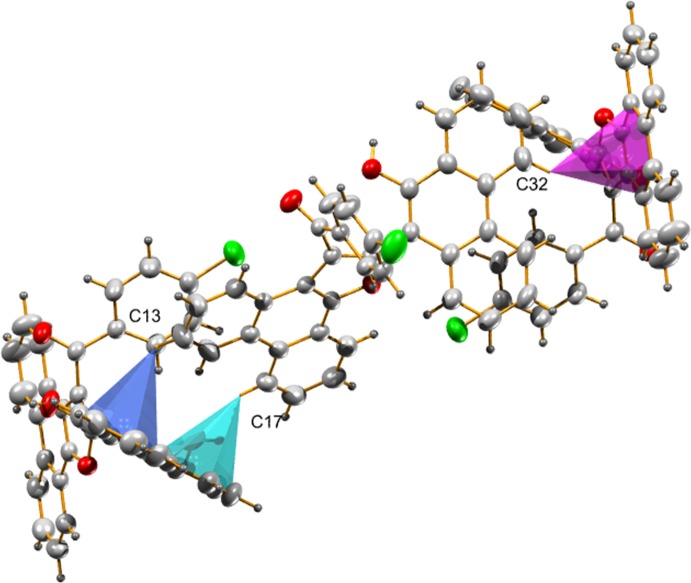
C—H⋯π inter­actions found in the title compound.

**Figure 4:**
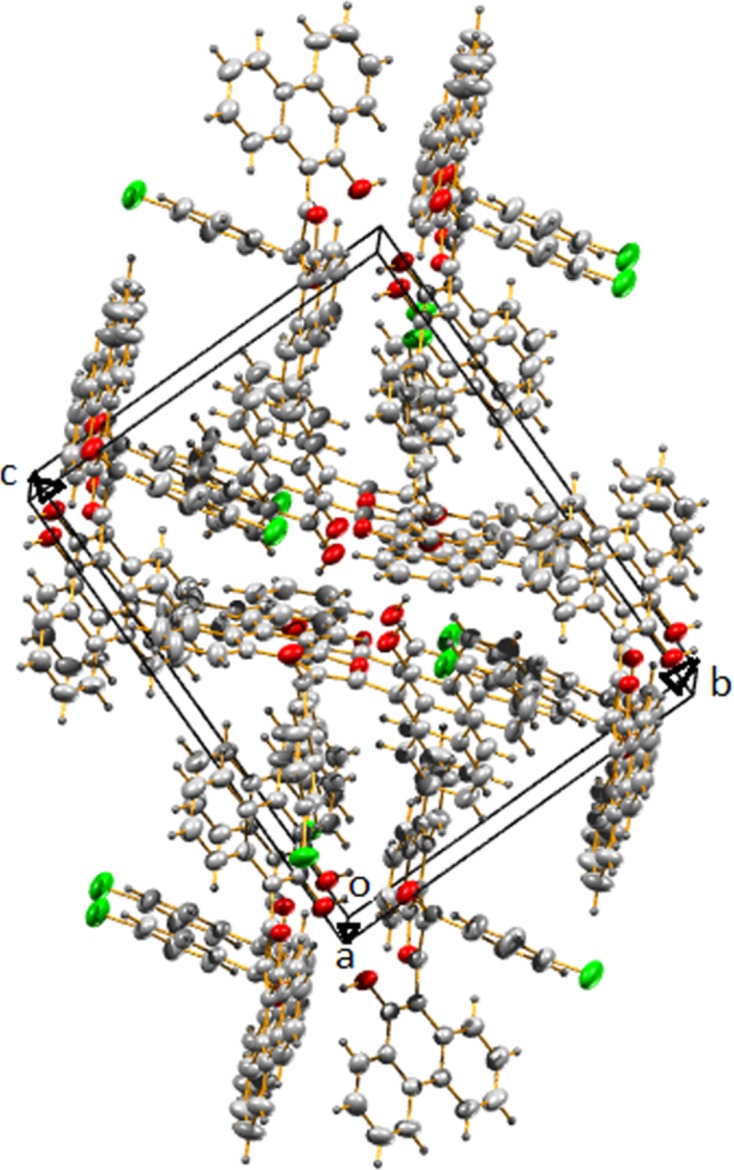
Packing diagram of the title compound along the *a* axis.

There are several inter­molecular hydrogen-bonding inter­actions present in the mol­ecular crystal. Carbonyl atom O1 acts as an acceptor for three hydrogen bonds; the intra­molecular C—H⋯O hydrogen bond with the H atom attached to C11, see above, and two inter­molecular hydrogen bonds involving atoms O3 and C35 of a neighbouring mol­ecule. The latter two inter­molecular hydrogen-bonding inter­actions lead to formation of an inversion dimer. Another non-classical hydrogen-bonding inter­action with the Cl atom of a neighbouring molecule as the acceptor connects these dimers, forming zigzag chains propagating in the *b*-axis direction (Fig. 2 ▶). Three C—H⋯π inter­actions (Fig. 3 ▶) are found in the crystal. The first two C—H⋯π inter­actions are between the H atoms attached to C13 and C17 and the outer two aromatic rings of one of the phenanthrene moieties of an adjacent mol­ecule with C⋯π distances of 3.709 (3) and 3.745 (2) Å. The third C—H⋯π inter­action occurs between atom C32 and the central aromatic ring of the other phenanthrene moiety (see Table 1 ▶ for numerical values and symmetry operators of O—H⋯O, C—H⋯O and C—H⋯π inter­actions). Fig. 4 ▶ shows the packing diagram of the title compound along *a* axis.

## Synthesis and crystallization   

**Figure 5:**
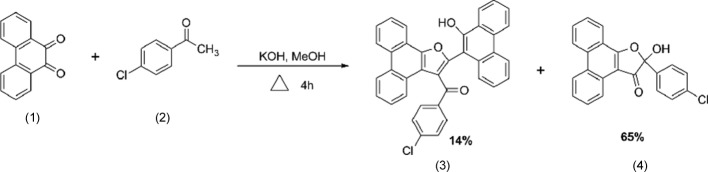
Reaction scheme showing the synthesis of the title compound (3).

A mixture of phenanthrene­quinone (1) (5.2 g, 25 mmol), 4-chloro­aceto­phenone (2) (4.2 g, 27 mmol) and powdered potassium hydroxide (1 g) in methanol (30 ml) was stirred at 333 K for 4 h and then kept in a refrigerator for 48 h. The main product obtained was a 3(2*H*)-furan­one [2-(4-chloro­phen­yl)-2-hy­droxy-1-oxa­cyclo­penta­[*l*]phenanthren-3-one] (4) (65%), which was purified by recrystallization from a mixture of methanol and di­chloro­methane (2:1 *v*/*v*). The title compound (3) was the minor product formed along with (4) during the reaction (Fig. 5 ▶). The reaction mixture was filtered and the filtrate was concentrated and subjected to column chromatography over silica gel. The title compound (14%) was separated on elution with a mixture of hexane and ethyl acetate (2:3 *v*/*v*). Diffraction-quality single crystals were generated by slow evaporation from methanol. Yield 1.90 g (14%); m.p. 459 K; IR (KBr, ν_max_): 3374 (OH), 1591 (C=O) cm^−1^; ^1^H NMR (CDCl_3_): δ 8.79–7.26 (*m*, 20H), 8.69 (*s*, 1H); MS: *m*/*z* 548 (*M* ^+^). Analysis calculated for C_37_H_21_ClO_3_: C 80.94, H 3.86%; found: C 80.82, H 3.66%.

## Refinement   

Crystal data, data collection and structure refinement details are summarized in Table 2 ▶. The C-bound H atoms were placed in calculated positions and treated as riding with C—H = 0.93 Å and with *U*_iso_(H) = 1.2*U*_eq_(C). The phenanthroline atom H3 was located from a difference Fourier map and refined with a distance restraint of O—H = 0.86 (1) Å. The reflection 101 was omitted owing to bad agreement. 

## Supplementary Material

Crystal structure: contains datablock(s) 3. DOI: 10.1107/S1600536814022338/zl2597sup1.cif

Structure factors: contains datablock(s) 3. DOI: 10.1107/S1600536814022338/zl25973sup2.hkl

CCDC reference: 1024475

Additional supporting information:  crystallographic information; 3D view; checkCIF report

## Figures and Tables

**Table 1:** Hydrogen-bond geometry (, ) *Cg*1 is the centroid of the C31C36 ring, *Cg*2 is the centroid of the C25C30 ring and Cg3 is the centroid of the C9/C10/C15/C16/C21/C22 ring.

*D*H*A*	*D*H	H*A*	*D* *A*	*D*H*A*
O3H3O1^i^	0.86(1)	2.01(2)	2.824(2)	156(3)
C20H20Cl1^ii^	0.93	2.68	3.564(2)	158
C35H35O1^i^	0.93	2.52	3.277(3)	139
C11H11O1	0.93	2.53	3.275(3)	137
C26H26O2	0.93	2.52	3.057(3)	117
C13H13*Cg*1^iii^	0.93	3.00	3.709(3)	134
C17H17*Cg*2^iii^	0.93	2.94	3.745(2)	146
C32H32*Cg*3^ii^	0.93	2.92	3.628(3)	134

Symmetry codes: (i) 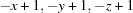; (ii) 
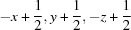
; (iii) 
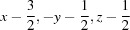
.

**Table 2:** Experimental details

Crystal data	
Chemical formula	C_37_H_21_ClO_3_
*M*_r_	548.99
Crystal system, space group	Monoclinic, *P*2_1_/*n*
Temperature (K)	296
*a*, *b*, *c* ()	11.6682(12), 13.4448(15), 17.071(2)
()	93.091(5)
*V* (^3^)	2674.1(5)
*Z*	4
Radiation type	Mo *K*
(mm^1^)	0.18
Crystal size (mm)	0.40 0.35 0.30

Data collection	
Diffractometer	Bruker Kappa APEXII CCD
Absorption correction	Multi-scan (*SADABS*; Bruker, 2007 ▶)
*T*_min_, *T*_max_	0.918, 0.920
No. of measured, independent and observed [*I* > 2(*I*)] reflections	19672, 5790, 3869
*R*_int_	0.027
(sin /)_max_ (^1^)	0.639

Refinement	
*R*[*F* ^2^ > 2(*F* ^2^)], *wR*(*F* ^2^), *S*	0.046, 0.147, 1.01
No. of reflections	5790
No. of parameters	374
No. of restraints	1
H-atom treatment	H atoms treated by a mixture of independent and constrained refinement
_max_, _min_ (e ^3^)	0.28, 0.36

Computer programs: *APEX2*, *SAINT* and *XPREP* (Bruker, 2007 ▶), *SHELXS2012* and *SHELXL97*(Sheldrick, 2008 ▶), *ORTEP-3 for Windows* (Farrugia, 2012 ▶), *DIAMOND* (Brandenburg, 2010 ▶), and *publCIF* (Westrip, 2010 ▶).

## supplementary crystallographic information

### Crystal data

**Table:**

C_37_H_21_ClO_3_	*F*(000) = 1136
*M**_r_* = 548.99	*D*_x_ = 1.364 Mg m^−^^3^
Monoclinic, *P*2_1_/*n*	Mo *K*α radiation, λ = 0.71073 Å
*a* = 11.6682 (12) Å	Cell parameters from 6603 reflections
*b* = 13.4448 (15) Å	θ = 2.4–27.5°
*c* = 17.071 (2) Å	µ = 0.18 mm^−^^1^
β = 93.091 (5)°	*T* = 296 K
*V* = 2674.1 (5) Å^3^	Block, yellow
*Z* = 4	0.40 × 0.35 × 0.30 mm

### Data collection

**Table:**

Bruker Kappa APEXII CCD diffractometer	3869 reflections with *I* > 2σ(*I*)
Detector resolution: 8.33 pixels mm^-1^	*R*_int_ = 0.027
ω and φ scan	θ_max_ = 27.0°, θ_min_ = 2.3°
Absorption correction: multi-scan (*SADABS*; Bruker, 2007)	*h* = −10→14
*T*_min_ = 0.918, *T*_max_ = 0.920	*k* = −16→17
19672 measured reflections	*l* = −21→21
5790 independent reflections	

### Refinement

**Table:**

Refinement on *F*^2^	1 restraint
Least-squares matrix: full	Hydrogen site location: mixed
*R*[*F*^2^ > 2σ(*F*^2^)] = 0.046	H atoms treated by a mixture of independent and constrained refinement
*wR*(*F*^2^) = 0.147	*w* = 1/[σ^2^(*F*_o_^2^) + (0.0706*P*)^2^ + 0.9152*P*] where *P* = (*F*_o_^2^ + 2*F*_c_^2^)/3
*S* = 1.01	(Δ/σ)_max_ < 0.001
5790 reflections	Δρ_max_ = 0.28 e Å^−^^3^
374 parameters	Δρ_min_ = −0.36 e Å^−^^3^

### Special details

**Table:**

Geometry. All e.s.d.'s (except the e.s.d. in the dihedral angle between two l.s. planes) are estimated using the full covariance matrix. The cell e.s.d.'s are taken into account individually in the estimation of e.s.d.'s in distances, angles and torsion angles; correlations between e.s.d.'s in cell parameters are only used when they are defined by crystal symmetry. An approximate (isotropic) treatment of cell e.s.d.'s is used for estimating e.s.d.'s involving l.s. planes.

### Fractional atomic coordinates and isotropic or equivalent isotropic displacement parameters (Å^2^)

**Table:**

	*x*	*y*	*z*	*U*_iso_*/*U*_eq_	
C1	0.40391 (18)	0.20554 (15)	0.31866 (12)	0.0457 (5)	
H1	0.3254	0.2178	0.3152	0.055*	
C2	0.4531 (2)	0.15068 (17)	0.26111 (13)	0.0518 (5)	
H2	0.4082	0.1250	0.2192	0.062*	
C3	0.5692 (2)	0.13462 (17)	0.26653 (13)	0.0522 (5)	
C4	0.6369 (2)	0.1693 (2)	0.32875 (15)	0.0642 (7)	
H4	0.7153	0.1566	0.3320	0.077*	
C5	0.58738 (18)	0.22282 (19)	0.38599 (13)	0.0562 (6)	
H5	0.6328	0.2463	0.4285	0.067*	
C6	0.47072 (16)	0.24266 (14)	0.38182 (11)	0.0390 (4)	
C7	0.42073 (16)	0.29570 (15)	0.44766 (11)	0.0391 (4)	
C8	0.29803 (16)	0.32528 (14)	0.44139 (11)	0.0383 (4)	
C9	0.20746 (16)	0.29570 (14)	0.49057 (11)	0.0367 (4)	
C10	0.19633 (17)	0.22173 (14)	0.55080 (11)	0.0397 (4)	
C11	0.28787 (19)	0.16154 (17)	0.57835 (14)	0.0531 (6)	
H11	0.3595	0.1690	0.5576	0.064*	
C12	0.2732 (2)	0.09222 (19)	0.63515 (17)	0.0680 (7)	
H12	0.3348	0.0527	0.6528	0.082*	
C13	0.1672 (2)	0.0801 (2)	0.66681 (18)	0.0751 (8)	
H13	0.1581	0.0334	0.7062	0.090*	
C14	0.0758 (2)	0.13712 (19)	0.64004 (15)	0.0634 (7)	
H14	0.0049	0.1281	0.6616	0.076*	
C15	0.08630 (17)	0.20866 (15)	0.58108 (12)	0.0439 (5)	
C16	−0.01135 (17)	0.26868 (15)	0.55198 (11)	0.0410 (4)	
C17	−0.12259 (18)	0.25754 (17)	0.57918 (13)	0.0516 (5)	
H17	−0.1354	0.2092	0.6167	0.062*	
C18	−0.21172 (19)	0.31559 (18)	0.55206 (14)	0.0558 (6)	
H18	−0.2839	0.3067	0.5716	0.067*	
C19	−0.19613 (19)	0.38775 (18)	0.49571 (14)	0.0535 (6)	
H19	−0.2572	0.4277	0.4780	0.064*	
C20	−0.09032 (18)	0.39981 (16)	0.46640 (12)	0.0466 (5)	
H20	−0.0798	0.4476	0.4281	0.056*	
C21	0.00207 (16)	0.34092 (14)	0.49349 (11)	0.0379 (4)	
C22	0.11395 (16)	0.34856 (14)	0.46446 (11)	0.0367 (4)	
C23	0.25215 (16)	0.39259 (15)	0.38951 (11)	0.0393 (4)	
C24	0.30128 (16)	0.45162 (14)	0.32712 (11)	0.0385 (4)	
C25	0.25286 (17)	0.44683 (15)	0.24773 (11)	0.0406 (4)	
C26	0.16075 (18)	0.38356 (17)	0.22663 (13)	0.0506 (5)	
H26	0.1304	0.3432	0.2646	0.061*	
C27	0.1146 (2)	0.38008 (19)	0.15127 (14)	0.0590 (6)	
H27	0.0536	0.3375	0.1386	0.071*	
C28	0.1583 (2)	0.4396 (2)	0.09386 (14)	0.0630 (6)	
H28	0.1259	0.4379	0.0429	0.076*	
C29	0.2489 (2)	0.50093 (18)	0.11228 (13)	0.0567 (6)	
H29	0.2783	0.5399	0.0731	0.068*	
C30	0.29902 (17)	0.50670 (15)	0.18884 (12)	0.0433 (5)	
C31	0.39590 (17)	0.57061 (15)	0.20959 (12)	0.0430 (5)	
C32	0.4470 (2)	0.63220 (18)	0.15423 (14)	0.0582 (6)	
H32	0.4176	0.6322	0.1025	0.070*	
C33	0.5380 (2)	0.69143 (19)	0.17483 (16)	0.0657 (7)	
H33	0.5692	0.7318	0.1372	0.079*	
C34	0.5849 (2)	0.69255 (18)	0.25086 (15)	0.0611 (6)	
H34	0.6471	0.7335	0.2644	0.073*	
C35	0.53931 (19)	0.63313 (17)	0.30604 (13)	0.0516 (5)	
H35	0.5719	0.6330	0.3570	0.062*	
C36	0.44394 (17)	0.57218 (15)	0.28718 (11)	0.0416 (5)	
C37	0.39348 (17)	0.51105 (15)	0.34560 (11)	0.0408 (4)	
O1	0.47866 (12)	0.31117 (12)	0.50815 (9)	0.0543 (4)	
O2	0.13803 (11)	0.40874 (10)	0.40306 (7)	0.0401 (3)	
O3	0.43790 (14)	0.51167 (13)	0.42054 (8)	0.0555 (4)	
Cl1	0.63277 (7)	0.06692 (6)	0.19461 (4)	0.0826 (3)	
H3	0.479 (2)	0.5630 (16)	0.4332 (18)	0.099 (11)*	

### Atomic displacement parameters (Å^2^)

**Table:**

	*U*^11^	*U*^22^	*U*^33^	*U*^12^	*U*^13^	*U*^23^
C1	0.0406 (11)	0.0519 (12)	0.0441 (11)	−0.0030 (9)	−0.0032 (9)	0.0002 (9)
C2	0.0611 (14)	0.0545 (13)	0.0386 (11)	−0.0019 (11)	−0.0075 (10)	−0.0051 (9)
C3	0.0636 (15)	0.0546 (13)	0.0385 (11)	0.0101 (11)	0.0025 (10)	−0.0030 (9)
C4	0.0445 (13)	0.0922 (19)	0.0558 (14)	0.0109 (13)	0.0016 (11)	−0.0152 (13)
C5	0.0399 (12)	0.0809 (16)	0.0470 (13)	0.0004 (11)	−0.0050 (9)	−0.0157 (11)
C6	0.0388 (11)	0.0428 (11)	0.0352 (10)	−0.0024 (8)	0.0004 (8)	0.0008 (8)
C7	0.0388 (10)	0.0444 (11)	0.0339 (10)	−0.0028 (8)	0.0005 (8)	0.0020 (8)
C8	0.0378 (10)	0.0441 (11)	0.0329 (10)	−0.0011 (8)	0.0007 (8)	−0.0003 (8)
C9	0.0366 (10)	0.0405 (10)	0.0327 (9)	−0.0026 (8)	−0.0008 (8)	−0.0040 (7)
C10	0.0439 (11)	0.0379 (10)	0.0369 (10)	−0.0058 (8)	−0.0023 (8)	0.0002 (8)
C11	0.0457 (12)	0.0529 (13)	0.0601 (14)	−0.0026 (10)	−0.0046 (10)	0.0108 (10)
C12	0.0579 (15)	0.0615 (15)	0.0833 (19)	−0.0006 (12)	−0.0085 (13)	0.0300 (14)
C13	0.0690 (17)	0.0701 (17)	0.086 (2)	−0.0087 (14)	−0.0023 (15)	0.0397 (15)
C14	0.0563 (14)	0.0634 (15)	0.0707 (17)	−0.0102 (12)	0.0057 (12)	0.0228 (12)
C15	0.0464 (12)	0.0421 (11)	0.0428 (11)	−0.0081 (9)	−0.0003 (9)	0.0024 (8)
C16	0.0420 (11)	0.0433 (11)	0.0379 (10)	−0.0074 (9)	0.0026 (8)	−0.0044 (8)
C17	0.0476 (13)	0.0578 (13)	0.0501 (13)	−0.0111 (10)	0.0086 (10)	0.0020 (10)
C18	0.0404 (12)	0.0696 (15)	0.0581 (14)	−0.0078 (11)	0.0099 (10)	−0.0057 (12)
C19	0.0417 (12)	0.0598 (14)	0.0592 (14)	0.0051 (10)	0.0025 (10)	−0.0077 (11)
C20	0.0468 (12)	0.0471 (12)	0.0461 (12)	0.0031 (9)	0.0039 (9)	−0.0017 (9)
C21	0.0382 (10)	0.0403 (10)	0.0352 (10)	−0.0026 (8)	0.0026 (8)	−0.0065 (8)
C22	0.0415 (11)	0.0373 (10)	0.0313 (9)	−0.0025 (8)	0.0028 (8)	−0.0013 (7)
C23	0.0358 (10)	0.0459 (11)	0.0364 (10)	−0.0012 (8)	0.0025 (8)	−0.0010 (8)
C24	0.0383 (10)	0.0433 (11)	0.0342 (10)	0.0037 (8)	0.0040 (8)	0.0028 (8)
C25	0.0396 (10)	0.0449 (11)	0.0372 (10)	0.0083 (9)	0.0012 (8)	0.0009 (8)
C26	0.0470 (12)	0.0622 (14)	0.0423 (12)	−0.0011 (10)	−0.0015 (9)	0.0021 (10)
C27	0.0507 (13)	0.0736 (16)	0.0516 (14)	−0.0023 (12)	−0.0082 (10)	−0.0070 (12)
C28	0.0632 (15)	0.0834 (18)	0.0406 (13)	0.0033 (13)	−0.0123 (11)	0.0005 (12)
C29	0.0623 (14)	0.0685 (15)	0.0389 (12)	0.0073 (12)	−0.0002 (10)	0.0078 (10)
C30	0.0465 (11)	0.0464 (11)	0.0369 (11)	0.0115 (9)	0.0013 (8)	0.0032 (8)
C31	0.0473 (12)	0.0425 (11)	0.0394 (11)	0.0086 (9)	0.0060 (9)	0.0055 (8)
C32	0.0616 (15)	0.0648 (15)	0.0482 (13)	0.0038 (12)	0.0042 (11)	0.0201 (11)
C33	0.0688 (16)	0.0640 (15)	0.0654 (17)	−0.0054 (13)	0.0128 (13)	0.0249 (12)
C34	0.0632 (15)	0.0562 (14)	0.0649 (16)	−0.0125 (12)	0.0140 (12)	0.0021 (11)
C35	0.0547 (13)	0.0554 (13)	0.0454 (12)	−0.0070 (10)	0.0097 (10)	−0.0038 (10)
C36	0.0453 (11)	0.0417 (11)	0.0385 (11)	0.0030 (9)	0.0086 (9)	−0.0008 (8)
C37	0.0438 (11)	0.0456 (11)	0.0333 (10)	0.0032 (9)	0.0037 (8)	−0.0012 (8)
O1	0.0476 (9)	0.0749 (11)	0.0398 (8)	0.0036 (7)	−0.0046 (7)	−0.0107 (7)
O2	0.0392 (7)	0.0454 (7)	0.0361 (7)	0.0021 (6)	0.0048 (6)	0.0047 (6)
O3	0.0629 (10)	0.0691 (11)	0.0340 (8)	−0.0196 (8)	−0.0022 (7)	0.0013 (7)
Cl1	0.1005 (6)	0.0945 (5)	0.0529 (4)	0.0339 (4)	0.0043 (3)	−0.0198 (3)

### Geometric parameters (Å, º)

**Table:**

C1—C2	1.378 (3)	C19—C20	1.367 (3)
C1—C6	1.389 (3)	C19—H19	0.9300
C1—H1	0.9300	C20—C21	1.396 (3)
C2—C3	1.370 (3)	C20—H20	0.9300
C2—H2	0.9300	C21—C22	1.425 (3)
C3—C4	1.370 (3)	C22—O2	1.365 (2)
C3—Cl1	1.728 (2)	C23—O2	1.381 (2)
C4—C5	1.367 (3)	C23—C24	1.469 (3)
C4—H4	0.9300	C24—C37	1.363 (3)
C5—C6	1.385 (3)	C24—C25	1.441 (3)
C5—H5	0.9300	C25—C26	1.402 (3)
C6—C7	1.477 (3)	C25—C30	1.417 (3)
C7—O1	1.221 (2)	C26—C27	1.369 (3)
C7—C8	1.484 (3)	C26—H26	0.9300
C8—C23	1.356 (3)	C27—C28	1.384 (3)
C8—C9	1.441 (3)	C27—H27	0.9300
C9—C22	1.357 (3)	C28—C29	1.364 (3)
C9—C10	1.441 (3)	C28—H28	0.9300
C10—C11	1.401 (3)	C29—C30	1.405 (3)
C10—C15	1.420 (3)	C29—H29	0.9300
C11—C12	1.362 (3)	C30—C31	1.449 (3)
C11—H11	0.9300	C31—C36	1.410 (3)
C12—C13	1.386 (4)	C31—C32	1.412 (3)
C12—H12	0.9300	C32—C33	1.359 (4)
C13—C14	1.372 (4)	C32—H32	0.9300
C13—H13	0.9300	C33—C34	1.381 (4)
C14—C15	1.402 (3)	C33—H33	0.9300
C14—H14	0.9300	C34—C35	1.365 (3)
C15—C16	1.461 (3)	C34—H34	0.9300
C16—C21	1.408 (3)	C35—C36	1.405 (3)
C16—C17	1.410 (3)	C35—H35	0.9300
C17—C18	1.361 (3)	C36—C37	1.442 (3)
C17—H17	0.9300	C37—O3	1.354 (2)
C18—C19	1.385 (3)	O3—H3	0.864 (10)
C18—H18	0.9300		
			
C2—C1—C6	120.52 (19)	C19—C20—C21	120.5 (2)
C2—C1—H1	119.7	C19—C20—H20	119.7
C6—C1—H1	119.7	C21—C20—H20	119.7
C3—C2—C1	119.0 (2)	C20—C21—C16	120.84 (18)
C3—C2—H2	120.5	C20—C21—C22	123.34 (18)
C1—C2—H2	120.5	C16—C21—C22	115.82 (18)
C2—C3—C4	121.7 (2)	C9—C22—O2	111.56 (16)
C2—C3—Cl1	119.73 (17)	C9—C22—C21	125.70 (18)
C4—C3—Cl1	118.58 (18)	O2—C22—C21	122.69 (17)
C5—C4—C3	119.0 (2)	C8—C23—O2	110.23 (16)
C5—C4—H4	120.5	C8—C23—C24	132.70 (18)
C3—C4—H4	120.5	O2—C23—C24	116.99 (16)
C4—C5—C6	121.1 (2)	C37—C24—C25	120.54 (18)
C4—C5—H5	119.4	C37—C24—C23	118.86 (17)
C6—C5—H5	119.4	C25—C24—C23	120.60 (17)
C5—C6—C1	118.66 (19)	C26—C25—C30	118.55 (19)
C5—C6—C7	118.75 (18)	C26—C25—C24	121.64 (18)
C1—C6—C7	122.41 (17)	C30—C25—C24	119.81 (18)
O1—C7—C6	120.15 (17)	C27—C26—C25	121.3 (2)
O1—C7—C8	120.22 (18)	C27—C26—H26	119.4
C6—C7—C8	119.56 (16)	C25—C26—H26	119.4
C23—C8—C9	106.78 (17)	C26—C27—C28	120.3 (2)
C23—C8—C7	124.84 (17)	C26—C27—H27	119.8
C9—C8—C7	128.25 (17)	C28—C27—H27	119.8
C22—C9—C8	105.43 (17)	C29—C28—C27	119.8 (2)
C22—C9—C10	119.59 (17)	C29—C28—H28	120.1
C8—C9—C10	134.70 (18)	C27—C28—H28	120.1
C11—C10—C15	119.61 (18)	C28—C29—C30	121.8 (2)
C11—C10—C9	122.79 (18)	C28—C29—H29	119.1
C15—C10—C9	117.58 (17)	C30—C29—H29	119.1
C12—C11—C10	120.8 (2)	C29—C30—C25	118.3 (2)
C12—C11—H11	119.6	C29—C30—C31	122.72 (19)
C10—C11—H11	119.6	C25—C30—C31	119.02 (18)
C11—C12—C13	120.4 (2)	C36—C31—C32	117.3 (2)
C11—C12—H12	119.8	C36—C31—C30	120.26 (18)
C13—C12—H12	119.8	C32—C31—C30	122.4 (2)
C14—C13—C12	120.0 (2)	C33—C32—C31	121.5 (2)
C14—C13—H13	120.0	C33—C32—H32	119.2
C12—C13—H13	120.0	C31—C32—H32	119.2
C13—C14—C15	121.7 (2)	C32—C33—C34	120.9 (2)
C13—C14—H14	119.2	C32—C33—H33	119.5
C15—C14—H14	119.2	C34—C33—H33	119.5
C14—C15—C10	117.5 (2)	C35—C34—C33	119.5 (2)
C14—C15—C16	121.72 (19)	C35—C34—H34	120.2
C10—C15—C16	120.77 (18)	C33—C34—H34	120.2
C21—C16—C17	116.52 (19)	C34—C35—C36	121.1 (2)
C21—C16—C15	120.50 (17)	C34—C35—H35	119.5
C17—C16—C15	122.98 (19)	C36—C35—H35	119.5
C18—C17—C16	121.8 (2)	C35—C36—C31	119.58 (19)
C18—C17—H17	119.1	C35—C36—C37	121.44 (19)
C16—C17—H17	119.1	C31—C36—C37	118.98 (18)
C17—C18—C19	120.7 (2)	O3—C37—C24	118.63 (17)
C17—C18—H18	119.6	O3—C37—C36	120.00 (18)
C19—C18—H18	119.6	C24—C37—C36	121.37 (18)
C20—C19—C18	119.5 (2)	C22—O2—C23	105.99 (14)
C20—C19—H19	120.2	C37—O3—H3	115 (2)
C18—C19—H19	120.2		
			
C6—C1—C2—C3	−1.0 (3)	C10—C9—C22—C21	2.5 (3)
C1—C2—C3—C4	1.9 (4)	C20—C21—C22—C9	177.58 (19)
C1—C2—C3—Cl1	−179.48 (17)	C16—C21—C22—C9	−2.9 (3)
C2—C3—C4—C5	−1.3 (4)	C20—C21—C22—O2	−5.4 (3)
Cl1—C3—C4—C5	−179.9 (2)	C16—C21—C22—O2	174.09 (16)
C3—C4—C5—C6	−0.2 (4)	C9—C8—C23—O2	0.7 (2)
C4—C5—C6—C1	1.1 (4)	C7—C8—C23—O2	−175.36 (17)
C4—C5—C6—C7	176.4 (2)	C9—C8—C23—C24	177.5 (2)
C2—C1—C6—C5	−0.4 (3)	C7—C8—C23—C24	1.5 (3)
C2—C1—C6—C7	−175.59 (19)	C8—C23—C24—C37	−55.3 (3)
C5—C6—C7—O1	−8.4 (3)	O2—C23—C24—C37	121.35 (19)
C1—C6—C7—O1	166.7 (2)	C8—C23—C24—C25	124.7 (2)
C5—C6—C7—C8	174.80 (19)	O2—C23—C24—C25	−58.6 (2)
C1—C6—C7—C8	−10.1 (3)	C37—C24—C25—C26	177.61 (19)
O1—C7—C8—C23	117.9 (2)	C23—C24—C25—C26	−2.4 (3)
C6—C7—C8—C23	−65.3 (3)	C37—C24—C25—C30	−2.0 (3)
O1—C7—C8—C9	−57.2 (3)	C23—C24—C25—C30	177.96 (17)
C6—C7—C8—C9	119.5 (2)	C30—C25—C26—C27	−1.0 (3)
C23—C8—C9—C22	−0.4 (2)	C24—C25—C26—C27	179.4 (2)
C7—C8—C9—C22	175.41 (19)	C25—C26—C27—C28	−0.1 (4)
C23—C8—C9—C10	173.2 (2)	C26—C27—C28—C29	1.0 (4)
C7—C8—C9—C10	−11.0 (3)	C27—C28—C29—C30	−0.9 (4)
C22—C9—C10—C11	177.33 (19)	C28—C29—C30—C25	−0.1 (3)
C8—C9—C10—C11	4.4 (3)	C28—C29—C30—C31	179.5 (2)
C22—C9—C10—C15	−0.9 (3)	C26—C25—C30—C29	1.0 (3)
C8—C9—C10—C15	−173.8 (2)	C24—C25—C30—C29	−179.31 (18)
C15—C10—C11—C12	−1.5 (3)	C26—C25—C30—C31	−178.58 (18)
C9—C10—C11—C12	−179.7 (2)	C24—C25—C30—C31	1.1 (3)
C10—C11—C12—C13	−0.1 (4)	C29—C30—C31—C36	−178.97 (19)
C11—C12—C13—C14	1.1 (5)	C25—C30—C31—C36	0.6 (3)
C12—C13—C14—C15	−0.3 (4)	C29—C30—C31—C32	0.2 (3)
C13—C14—C15—C10	−1.3 (4)	C25—C30—C31—C32	179.77 (19)
C13—C14—C15—C16	179.4 (2)	C36—C31—C32—C33	−0.7 (3)
C11—C10—C15—C14	2.2 (3)	C30—C31—C32—C33	−179.8 (2)
C9—C10—C15—C14	−179.59 (19)	C31—C32—C33—C34	0.8 (4)
C11—C10—C15—C16	−178.45 (19)	C32—C33—C34—C35	0.2 (4)
C9—C10—C15—C16	−0.2 (3)	C33—C34—C35—C36	−1.3 (4)
C14—C15—C16—C21	179.1 (2)	C34—C35—C36—C31	1.4 (3)
C10—C15—C16—C21	−0.2 (3)	C34—C35—C36—C37	−178.4 (2)
C14—C15—C16—C17	−1.7 (3)	C32—C31—C36—C35	−0.4 (3)
C10—C15—C16—C17	178.98 (19)	C30—C31—C36—C35	178.76 (19)
C21—C16—C17—C18	−1.9 (3)	C32—C31—C36—C37	179.40 (18)
C15—C16—C17—C18	178.9 (2)	C30—C31—C36—C37	−1.4 (3)
C16—C17—C18—C19	0.6 (3)	C25—C24—C37—O3	−178.54 (18)
C17—C18—C19—C20	0.8 (3)	C23—C24—C37—O3	1.5 (3)
C18—C19—C20—C21	−0.8 (3)	C25—C24—C37—C36	1.2 (3)
C19—C20—C21—C16	−0.6 (3)	C23—C24—C37—C36	−178.75 (17)
C19—C20—C21—C22	178.89 (19)	C35—C36—C37—O3	0.1 (3)
C17—C16—C21—C20	1.9 (3)	C31—C36—C37—O3	−179.74 (18)
C15—C16—C21—C20	−178.83 (18)	C35—C36—C37—C24	−179.69 (19)
C17—C16—C21—C22	−177.64 (18)	C31—C36—C37—C24	0.5 (3)
C15—C16—C21—C22	1.6 (3)	C9—C22—O2—C23	0.4 (2)
C8—C9—C22—O2	0.0 (2)	C21—C22—O2—C23	−177.00 (17)
C10—C9—C22—O2	−174.73 (16)	C8—C23—O2—C22	−0.6 (2)
C8—C9—C22—C21	177.31 (17)	C24—C23—O2—C22	−178.04 (16)

### Hydrogen-bond geometry (Å, º)

Cg1 is the centroid of the C31–C36 ring, Cg2 is the centroid of the C25–C30 ring and Cg3 is the centroid of the C9/C10/C15/C16/C21/C22 ring.

**Table:**

*D*—H···*A*	*D*—H	H···*A*	*D*···*A*	*D*—H···*A*
O3—H3···O1^i^	0.86 (1)	2.01 (2)	2.824 (2)	156 (3)
C20—H20···Cl1^ii^	0.93	2.68	3.564 (2)	158
C35—H35···O1^i^	0.93	2.52	3.277 (3)	139
C11—H11···O1	0.93	2.53	3.275 (3)	137
C26—H26···O2	0.93	2.52	3.057 (3)	117
C13—H13···*Cg*1^iii^	0.93	3.00	3.709 (3)	134
C17—H17···*Cg*2^iii^	0.93	2.94	3.745 (2)	146
C32—H32···*Cg*3^ii^	0.93	2.92	3.628 (3)	134

Symmetry codes: (i) −*x*+1, −*y*+1, −*z*+1; (ii) −*x*+1/2, *y*+1/2, −*z*+1/2; (iii) *x*−3/2, −*y*−1/2, *z*−1/2.
